# Endothelial GPR68 Is Essential for Arteriogenesis and Represents a Therapeutic Target in a Model of Peripheral Artery Disease

**DOI:** 10.1002/advs.202520244

**Published:** 2026-03-20

**Authors:** Yiyan Song, Senyi Peng, Zhilei Huang, Miao Yu, Mingyue Wu, Yuexin Zhu, Zeyi Zhou, Yongshan Liu, Yalin Zhang, Yu Huang, Zhongxing Wang, Fu‐Li Xiang, Jie Xu

**Affiliations:** ^1^ Department of Anesthesia The First Affiliated Hospital of Sun Yat‐Sen University Guangzhou China; ^2^ Institute of Precision Medicine The First Affiliated Hospital of Sun Yat‐Sen University Guangzhou China; ^3^ Interventional Center of Valvular Heart Disease Beijing Anzhen Hospital Capital Medical University Beijing China; ^4^ Department of Cardiovascular Surgery Nanjing Drum Tower Hospital Affiliated Hospital of Medical School Nanjing China; ^5^ Department of Biomedical Sciences City University of Hong Kong Hong Kong China; ^6^ NHC Key Laboratory of Assisted Circulation and Vascular Diseases Sun Yat‐sen University Guangzhou China

**Keywords:** Arteriogenesis, GPR68, Hindlimb Ischemia (HLI), Peripheral Artery Diseases

## Abstract

Peripheral artery disease (PAD) is characterized by arterial narrowing that reduces limb perfusion, causing significant morbidity. The body can compensate via shear stress‐driven collateral artery growth (arteriogenesis). However, the primary receptors of mechanical forces on the endothelial cells in PAD remain poorly defined. Human phenome‑wide association study (PheWAS) shows that variants near GPR68, a gene encoding shear‐stress sensitive G protein‐coupled receptor (GPCR), are associated with increased PAD risk. To investigate the role of GPR68 in arteriogenesis in PAD, hindlimb ischemia (HLI) was employed in inducible, endothelial cell‐specific *Gpr68* knockout mice (Gpr68 iECKO) as well as littermate controls. Impaired blood perfusion recovery, smaller collateral artery diameter, and exacerbated distal muscle injury were observed in Gpr68 iECKO. Mechanistically, these defects were associated with a reduction in perivascular CCR2^+^ macrophage recruitment, linked to the downregulation of *Spp1*, *Ccl2*, and *Itgb3*. Pharmacological activation of Gpr68 with its agonist Compound 71 enhances monocyte adhesion to ECs via increased SPP1 in vitro. In vivo, Compound 71 accelerates perfusion recovery and mitigates ischemic tissue damage. Our findings establish that endothelial GPR68 is a critical mediator of the inflammatory‐remodeling program essential for arteriogenesis. We propose that pharmacological activation of GPR68 represents a novel therapeutic strategy for PAD.

## Graphical abstract

Endothelial GPR68 is identified as a critical regulator of collateral artery growth in peripheral artery disease. Genetic and pharmacological evidence demonstrates that GPR68 integrates hemodynamic cues to drive monocyte recruitment and inflammatory remodeling, thereby promoting collateral arteriogenesis and tissue perfusion after ischemia, highlighting GPR68 as a potential therapeutic target for ischemic vascular disease.

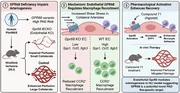

## 1 Introduction

Peripheral artery disease (PAD) stems from the pathological narrowing of limb arteries, which compromises blood flow to distal tissues [1]. The clinical spectrum ranges from intermittent claudication to critical limb ischemia, which carries a high risk of amputation [2]. A key determinant of patient outcomes is the capacity for collateral vessel growth, which can mitigate ischemia and reduce tissue loss. Augmenting this natural process, known as arteriogenesis, is a promising therapeutic goal [3].

Arteriogenesis involves the remodeling of pre‐existing arterioles into larger arteries. Unlike angiogenesis, it is initiated by physical and inflammatory signals [4, 5]. Following an arterial occlusion, rerouted blood flow dramatically increases fluid shear stress on the endothelium of collateral vessels. This mechanical stimulus is a key trigger, causing the endothelium to adopt a pro‐inflammatory and adhesive phenotype that is essential for recruiting monocytes to the vessel wall [6, 7]. This recruitment process is orchestrated by a complex interplay of specific molecular regulators. A critical pathway involves the upregulation of Chemokine (C─C motif) ligand 2 (CCL2) by endothelial cells, which acts as a potent chemoattractant for circulating monocytes that express its receptor, CCR2 [8]. In parallel, the expression of various adhesion‐related molecules is increased to facilitate the capture and firm binding of these leukocytes. Key players in this context include Secreted Phosphoprotein 1 (SPP1), a cytokine that binds to Integrin Subunit Beta 3 (ITGB3) and promotes monocyte and endothelial cell–cell adhesion [9]. The successful recruitment and perivascular accumulation of monocytes is the defining step that initiates the structural remodeling of the vessel [10]. However, although several shear stress‐regulated proteins have been identified in mouse hindlimb ischemia (HLI) models, such as PECAM1 [11, 12], glycocalyx [13] and integrin α5 [14], the specific endothelial mechanosensor responsible for collateral vessel growth in PAD that translates physical shear stress signal to the first event in the signal transduction pathway, such as ionic flux or electrical currents remains poorly defined.

G protein‐coupled receptor 68 (GPR68), also known as Ovarian Cancer G‐protein coupled Receptor 1 (OGR1), has been identified as a receptor capable of sensing multiple stimuli [15, 16]. We and others have shown that GPR68 can be activated by shear stress, membrane stretch, and substrate stiffness, often requiring a permissive pH range to function [17, 18, 19]. While GPR68 has been well‐characterized as a proton sensor in the context of cerebral ischemia, where tissue acidosis is a hallmark of stagnant infarcts, its role in the peripheral vasculature appears to be driven by mechanical cues. In the hindlimb, the diversion of blood through collateral circuits subjects the endothelium to significant increases in fluid shear stress [20, 21], suggesting that GPR68 may serve a context‐dependent role as a mechanosensor during vascular remodeling. Our previous work has shown that genetic loss of Gpr68 impairs both acute flow‐mediated dilation and chronic flow‐induced outward remodeling of mouse mesenteric arteries, implicating it in adaptive vascular responses. This function is consistent with its high expression in the endothelial cells of mesenteric small‐diameter resistance arteries [17]. Despite these crucial findings, its specific function in the context of post‐occlusive collateral artery growth in PAD has not been investigated. Here, we test the hypothesis that endothelial GPR68 is an essential sensor that drives arteriogenesis in a mouse model of PAD.

## 2 Results

### 2.1 Variants Near GPR68 Are Associated With Increased PAD Risk

**FIGURE 1:**
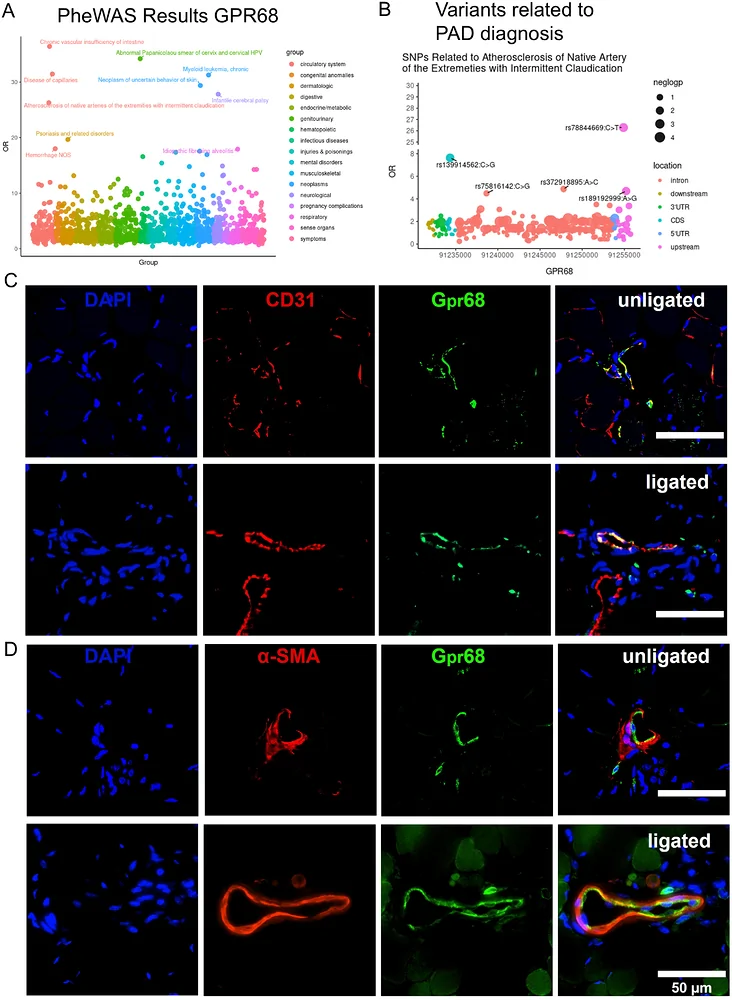
GPR68 is associated with PAD in humans and expressed in remodeling collateral arteries. (A) Phenome‐wide association study (PheWAS) plot showing associations between variants in the GPR68 locus and various disease categories from the UK Biobank. The *y*‐axis represents the odds ratio (OR), and points are colored by disease group, with circulatory system traits highlighted in red. (B) Locus plot showing single‐nucleotide variants across the GPR68 locus associated with peripheral artery disease (PAD). The y‐axis shows the odds ratio, and the point size corresponds to the statistical significance (−log10 (*p*‐value)). (C) Representative immunofluorescence images of collateral arterioles in the semimembranosus muscle from Gpr68‐tdTomato reporter mice. Images show Gpr68 expression (green, tdTomato signal), endothelial cells (red, CD31), and nuclei (blue, DAPI) in unligated (top) and ligated (bottom) hindlimbs. The merged image shows co‐localization of Gpr68 in endothelial cells. (D) Immunofluorescence images showing Gpr68 (green) and smooth muscle cells (red, α‐SMA) in collateral arterioles from Gpr68‐tdTomato reporter mice. Scale bars for D = 50 µm.

To explore whether human genetic variation implicates GPR68 in peripheral artery disease (PAD), we performed a phenome‐wide association study (PheWAS) across the GPR68 locus using UK Biobank data. At the phenotypic level, variants within the GPR68 locus showed enrichment for circulatory system traits, including atherosclerosis of the extremities and intermittent claudication, both core components of PAD (Figure 1A; Figure S1A). Variant‐level analysis focusing on PAD revealed multiple associated single‐nucleotide variants distributed across upstream, 5’‐UTR, and intronic regions of the GPR68 locus (Figure 1B). Effect sizes are shown as odds ratios, with point size reflecting statistical significance (−log10 (*p*‐alue)). Among these, an upstream non‐coding variant (rs78844669) exhibited the largest effect size. Notably, all PAD‐associated variants identified in this locus are non‐coding, and no coding or protein‐altering variants were detected.

These data indicate that genetic variation at the GPR68 locus is associated with PAD susceptibility at the population level. However, because the associated variants are non‐coding, these findings do not establish a causal mechanism, functional directionality (gain‐ or loss‐of‐function), or direct effects on GPR68 expression. The identified non‐coding variants likely function as eQTLs (expression quantitative trait loci) that modulate GPR68 expression levels. The human genetic association implicates the GPR68 locus in PAD risk and provides a rationale for subsequent mechanistic investigation.

### 2.2 Gpr68 is Expressed in Arteriole Endothelial Cells of Remodeling Collateral Arteries and Infiltrating Myeloid Cells in Ischemic Tissue

To elucidate the cellular context of GPR68 in the setting of HLI, due to the lack of a validated GPR68 antibody, we investigated its expression pattern using *Gpr68*‐*tdTomato* reporter mice (Figure S1B). Consistent with our previous findings, we observed tdTomato signal specifically within CD31‐positive endothelial cells lining the collateral arterioles of the semimembranosus muscle in both unligated and ligated hind limbs (Figure 1C,D), but no expression of GPR68 was found in capillaries and veins (Figure S1C). At baseline, very few macrophages were found around the arterioles (Figure S1D). However, seven days after HLI, significantly increased F4/80+ macrophages were observed around the arterioles, the majority of which were also GPR68‐positive (Figure S1D). No neutrophils were found around the arterioles at this time point (Figure S1E). In the more distal, ischemic gastrocnemius muscle, the density of GPR68‐positive cells majorly co‐expressing the immune cell markers CD45 dramatically increased following HLI (Figure S1F). These observations indicate that after HLI, GPR68 is predominantly expressed by two cell populations critical to vascular repair: the endothelial cells of small‐diameter, high‐shear collateral vessels and the newly recruited macrophages within ischemic tissue, positioning the receptor at a key intersection of vascular remodeling and inflammation.

### 2.3 Endothelial‐Specific Gpr68 Deletion Impairs Perfusion Recovery and Collateral Growth

**FIGURE 2:**
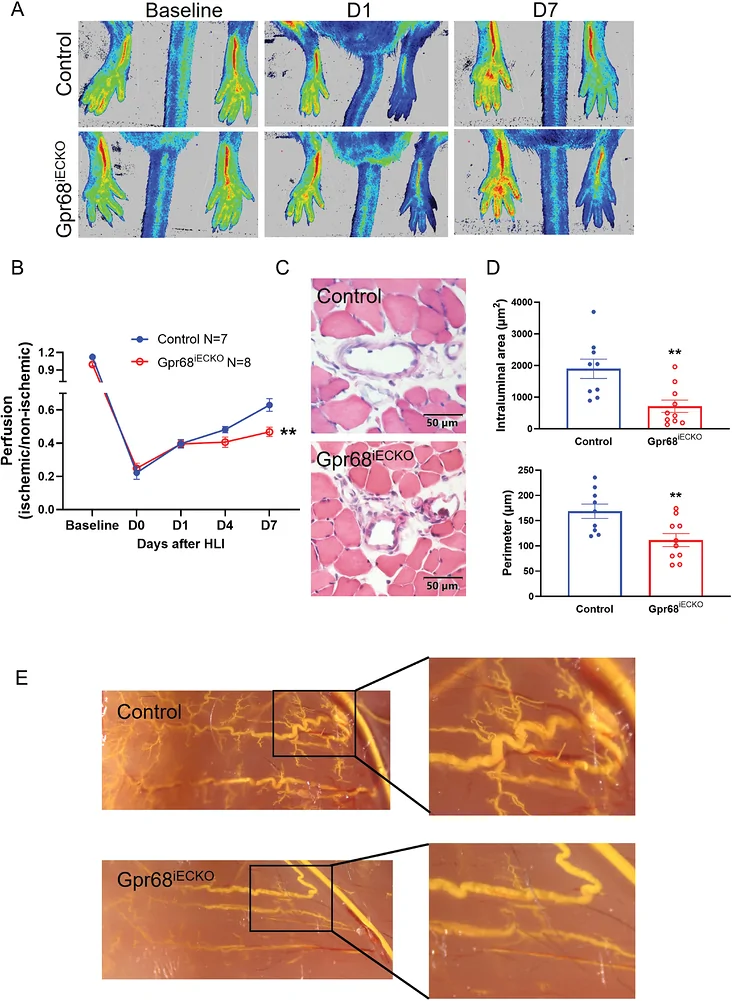
Endothelial‐specific deletion of Gpr68 impairs perfusion recovery and collateral growth after hindlimb ischemia. (A) Representative laser Doppler perfusion images of hindpaws from control and inducible, endothelial cell‐specific Gpr68 knockout (Gpr68 iECKO) mice at baseline, 1 day, and 7 days after hindlimb ischemia (HLI) surgery. (B) Quantification of blood perfusion recovery over 7 days, expressed as the ratio of ischemic (left) to non‐ischemic (right) limb perfusion. Gpr68 iECKO mice show significantly impaired recovery compared to controls. N = 7–8 mice per group, **p < 0.01 vs Control, analyzed by Student's t‐test. (C) Representative H&E‐stained cross‐sections of collateral arteries in the semimembranosus muscle 7 days post‐HLI. (D) Quantification of the intraluminal area and perimeter of collateral arteries, showing significantly smaller vessels in Gpr68 iECKO mice. N = 9–10 mice per group, **p < 0.01 vs Control, analyzed by Student's t‐test. (E) Representative images of collateral vasculature visualized by MICROFIL casting, demonstrating a blunted arteriogenic response in Gpr68 iECKO mice compared to controls. Images are presented as qualitative corroboration. Data are presented as mean ± SEM. Scale bars = 50 µm.

To dissect the functional contribution of endothelial GPR68, we subjected inducible, EC‐specific knockout mice (Gpr68 iECKO) to HLI. Laser Doppler imaging revealed that perfusion recovery in Gpr68 iECKO mice was significantly impaired compared to littermate controls at day 7 post‐surgery (Figure 2A,B). Histological analysis including H&E staining and CD31/αSMA staining (Figure S2A) confirmed a structural correlate for this functional deficit. Specifically, collateral morphometry was performed from 3 sections in the center part of semimembranosus muscle of the adductor muscle series [22] as it is a major component of the medial thigh/adductor compartment where pre‐existing arterioles reliably undergo outward remodeling into functional collateral arteries. Collateral arteries in the semimembranosus muscle of Gpr68 iECKO mice were significantly smaller, with a reduced intraluminal area and perimeter (Figure 2C,D). Furthermore, vascular casting with MICROFIL provided a striking visualization of a blunted arteriogenesis, showing a marked reduction in the number and tortuosity of collateral branches in Gpr68 iECKO mice (Figure 2E). In the context of the HLI model, increased tortuosity is a well‐established morphological indicator of active arteriogenesis [23, 24]. As pre‐existing collateral vessels undergo expansive outward remodeling in response to elevated shear stress, they increase in both diameter and longitudinal length. There is no difference in vessel tortuosity at baseline between Control and Gpr68 iECKO mice (Figure S2B). This data provides compelling evidence that endothelial GPR68 plays a critical role in the adaptive outward remodeling of collaterals required to restore blood flow after arterial occlusion in HLI.

### 2.4 Impaired Arteriogenesis in Gpr68 iECKO Mice Exacerbates Ischemic Muscle Injury

**FIGURE 3:**
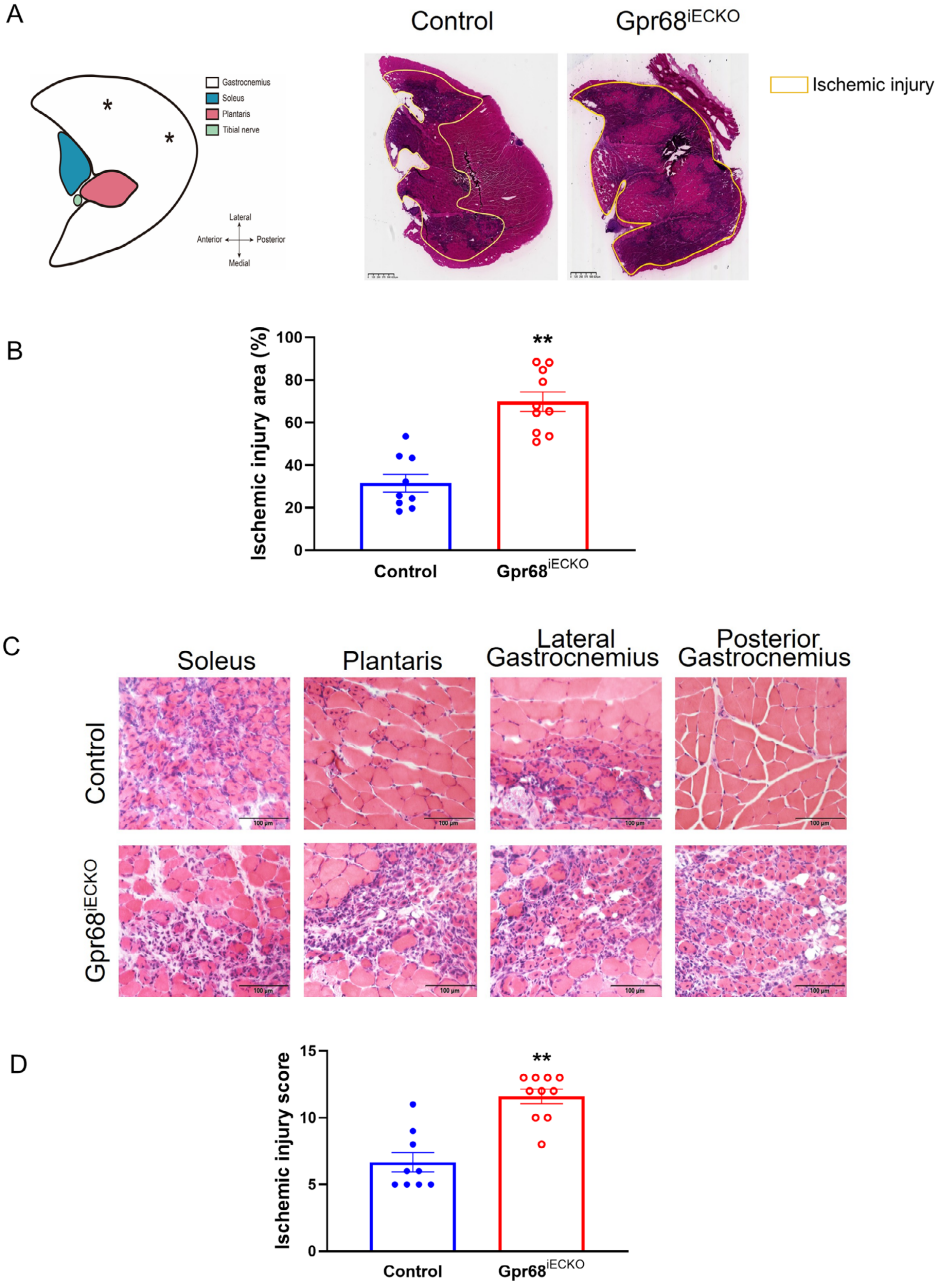
Impaired arteriogenesis in Gpr68 iECKO mice leads to exacerbated ischemic muscle injury. (A) Schematic of hindlimb muscle groups (left) and representative H&E‐stained cross‐sections of the gastrocnemius muscle from control and Gpr68 iECKO mice 7 days post‐HLI. The yellow outlined area indicates ischemic injury. (B) Quantification of the ischemic injury area, expressed as a percentage of the total muscle cross‐sectional area, showing significantly greater injury in knockout mice. N = 9–10 mice per group, **p < 0.01 vs Control, analyzed by Student's t‐test. (C) High‐magnification images of H&E‐stained sections from various distal muscles (Soleus, Plantaris, Lateral and Posterior Gastrocnemius) showing widespread muscle fiber necrosis and inflammatory infiltration in Gpr68 iECKO mice. (D) Quantification of ischemic injury using a histological scoring system. N = 9–10 mice per group, **p < 0.01 vs Control, analyzed by Mann–Whitney U test. Data are presented as mean ± SEM. Scale bars = 100 µm.

We next sought to determine if the defective collateral growth in Gpr68 iECKO mice resulted in more severe ischemic damage to the distal musculature. Histopathological analysis of the gastrocnemius muscle at day 7 post‐HLI revealed significantly exacerbated injury in Gpr68 iECKO mice compared to controls (Figure 3A,B). The knockout mice exhibited widespread muscle fiber necrosis, degeneration, and pronounced inflammatory leukocyte infiltration, whereas control limbs displayed only limited, focal damage (Figure 3C,D). This finding confirms that the impaired GPR68‐mediated arteriogenesis translates directly to more severe distal tissue injury.

### 2.5 Endothelial GPR68 Drives Arteriogenesis by Promoting Perivascular Monocyte Recruitment

**FIGURE 4:**
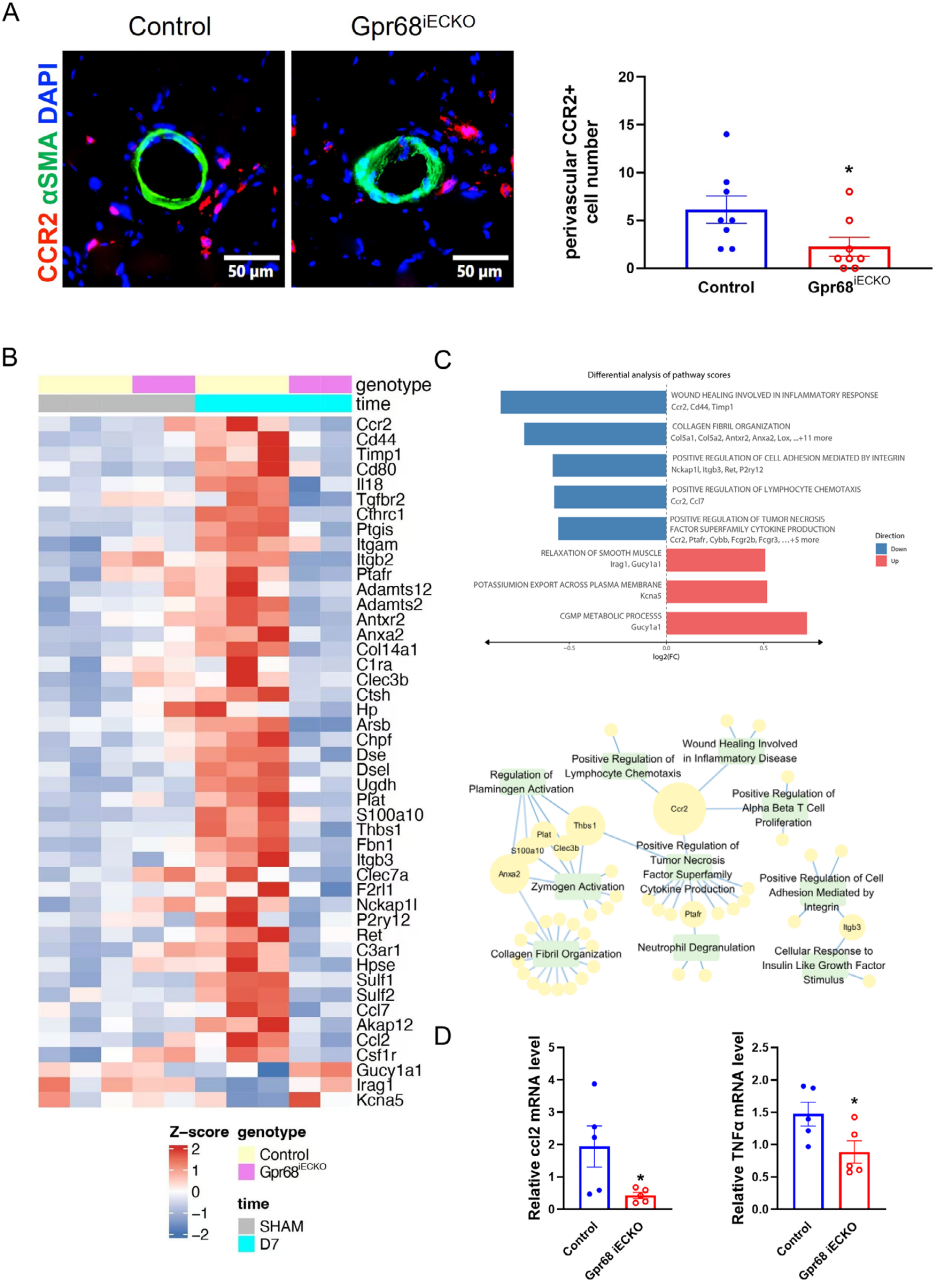
Endothelial Gpr68 deletion reduces perivascular monocyte recruitment and downregulates pro‐inflammatory gene expression. (A) Representative immunofluorescence images and quantification of CCR2^+^ cells (red) around collateral arteries (αSMA, green) in the semimembranosus muscle of control and Gpr68 iECKO mice 7 days post‐HLI. Nuclei are stained with DAPI (blue). CCR2 was used to selectively identify monocyte‐derived macrophages recruited from the circulation, as CCR2 distinguishes recruited inflammatory monocytes from resident macrophage populations. Perivascular CCR2^+^ monocyte/macrophage recruitment is significantly reduced in knockout mice. N = 8 mice per group, *p < 0.05 vs Control, analyzed by Student's t‐test. (B) Heatmap showing Z‐scores of differentially expressed genes from RNA sequencing of the adductor muscle region containing remodeling collaterals from wild‐type (WT) and knockout (KO) mice at day 7 (D7) post‐HLI or sham surgery. (C) Pathway‐gene network analysis of RNA‐seq data showing downregulation of pathways involved in monocyte recruitment, inflammation, TNF superfamily cytokine production, and cell adhesion in Gpr68 iECKO mice. (D) qPCR validation showing significantly lower mRNA expression of Ccl2 and TNFα in the collateral‐containing tissue of Gpr68 iECKO mice. N = 5 mice per group, *p < 0.05 vs Control, analyzed by Student's t‐test. Data are presented as mean ± SEM. Scale bars = 50 µm.

Arteriogenesis in HLI is initiated by monocyte recruitment via CCL2/CCR2 chemokine signaling in response to shear stress‐induced EC activation in the collateral vessel wall [25, 26]. To selectively quantify macrophages recruited from the circulation, we analyzed CCR2^+^ cells in the perivascular region of remodeling collaterals. CCR2 marks inflammatory monocytes required for arteriogenesis and distinguishes recruited monocyte‐derived macrophages from resident populations, which cannot be differentiated by CD68 or F4/80 alone. Immunofluorescence staining of semimembranosus collaterals showed that the number of perivascular CCR2^+^ monocytes/macrophages was reduced by approximately 50% in Gpr68 iECKO mice seven days after HLI (Figure 4A). Representative co‐staining for CCR2 with CD68 confirmed macrophage identity (Figure S2C). To define the molecular basis for this recruitment defect, we performed RNA‐sequencing on tissue from the adductor region containing the remodeling collaterals. Because this analysis was conducted on whole collateral‐containing muscle tissue, the resulting transcriptomic profiles reflect both transcriptional regulation and changes in cellular composition within the remodeling microenvironment, rather than endothelial‐intrinsic gene expression alone. This transcriptomic analysis revealed a coordinated downregulation of a gene set (Figure 4B) involved in monocyte recruitment, including key chemokines, adhesion molecules and TNFα‐associated signaling (Figure 4C), in Gpr68 iECKO mice. Based on the RNAseq top hits and associated pathways, subsequent qPCR validation confirmed that the expression of *Ccl2* and *Tnfα* was significantly lower in the tissue containing the remodeling collaterals in Gpr68 iECKO mice (Figure 4D), while no changes in *Icam*, *Vcam*, *Mmp9*, and *Nos3* were observed (Figure S2D). Validation of Gpr68 knockdown in Gpr68 iECKO and myeloid‐specific Gpr68 KO mice are shown in Figure S2E. These results indicate that endothelial Gpr68 signaling is required to generate the pro‐inflammatory and adhesive vascular environment necessary for monocyte recruitment and subsequent collateral growth.

### 2.6 Pharmacological GPR68 Activation Promotes Monocyte Adhesion In Vitro

**FIGURE 5:**
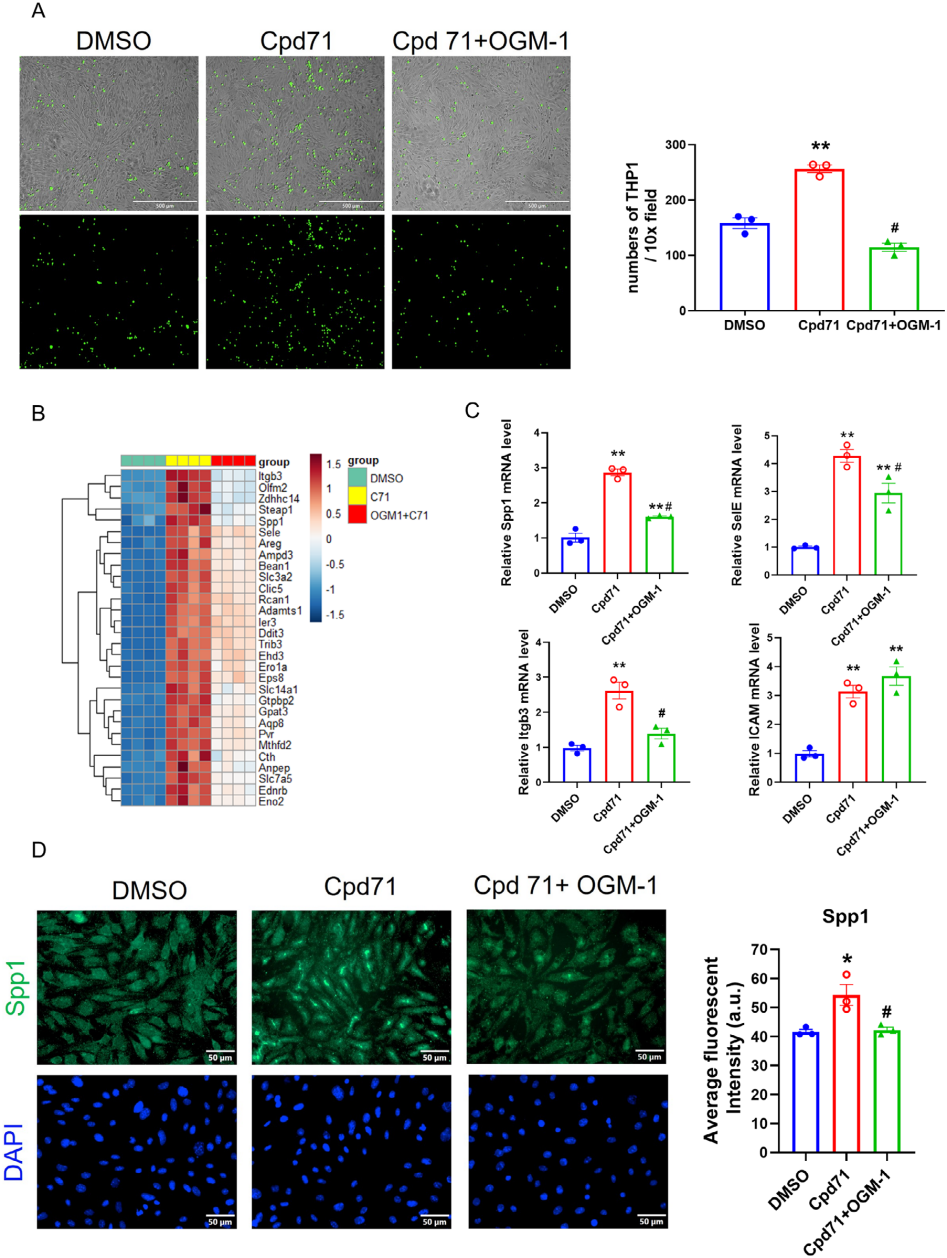
Pharmacological activation of Gpr68 promotes monocyte adhesion and a pro‐inflammatory endothelial phenotype in vitro. (A) Representative images and quantification of an in vitro adhesion assay showing increased binding of THP‐1 monocytes to endothelial cell monolayers pre‐treated with the Gpr68 agonist Compound 71 (Cpd71). This effect is blocked by the Gpr68 inhibitor OGM‐1. Scale bars = 500 µm. (B) Heatmap of bulk RNA‐seq data from endothelial cells treated with DMSO (vehicle), Cpd71, or Cpd71+OGM‐1, showing genes associated with monocyte adhesion are induced by Cpd71. (C) qPCR validation of key adhesion‐related genes (*Spp1*, *SelE*, *Itgb3*, *Icam*) showing Cpd71‐induced expression is GPR68‐dependent. Notably, Spp1 transcript levels were robustly induced by GPR68 agonist treatment (∼3‐fold increase), and this induction was reduced by approximately 50% upon co‐treatment with the GPR68 inhibitor OGM‐1. (D) Representative immunofluorescence images and quantification showing increased SPP1 protein expression (green) in endothelial cells following Cpd71 treatment, an effect reversed by OGM‐1. Nuclei are stained with DAPI (blue). Scale bars = 50 µm. Data are presented as mean ± SEM. N = 3. *p < 0.05 **p < 0.01 vs. DMSO group; #*p* < 0.05 vs. Cpd71 group, analyzed by one‐way ANOVA with Tukey's post‐hoc test.

Our previous study has demonstrated the role of GPR68 in shear stress induced mechanosensing in endothelial cells via Gq‐Ca^2+^ signaling [17]. Having established the necessity of endothelial Gpr68 in collateral arteriogenesis in HLI, we started seeking for GPR68's therapeutic potential through the reported GPR68‐specific agonistic allosteric modulator Compound 71 (Cpd71) [19]. We tested the specificity of Cpd71 on primary cultured brain ECs that partially express GPR68 (Figure S3A,B). Cpd71 triggered a rapid intracellular Ca^2+^ increase in wild‐type ECs, an effect that was completely absent in ECs (Figure S4C) in Gpr68 global KO (Figure S3D), confirming its target specificity. To determine the downstream pathway of Gpr68, commercially available mouse brain microvascular EC (bEnd.3) line was used. Our in vivo findings demonstrate that GPR68 expression is localized specifically to the endothelial cells of small‐diameter arterioles (collaterals) rather than large conduit arteries, veins, and capillaries [17] (Figure S1C). Because arteriogenesis in our model involves the remodeling of these small pre‐existing arterioles, we selected the mouse brain microvascular endothelial cell (bEnd.3) line, bEnd.3, as a representative model. Due to lack of validated Gpr68 antibody, Cpd71 induced calcium signal at 30 and 50 µm was used to confirm the Gpr68 expression in bEnd.3 (Figure S3E) but not in human HUVEC line (Figure S3F). In an adhesion assay, pre‐treatment of bEnd.3 monolayers with Cpd71 increased the binding of THP‐1 monocytes by 1.6‐fold (Figure 5A). No changes in cell proliferation, migration, and cell death were observed in Cpd71‐treated bEnd.3 (Figure S4A–E). The effects of Cpd71 on monocyte adhesion were abrogated by a Gpr68 inhibitor OGM‐1 [27] (Figure 5A). Bulk RNAseq from bEnd.3 treated with Cpd71 and Cpd71 plus OGM‐1 were performed. Top 30 gene candidates for Gpr68 downstream pathways are listed in Figure 5B, most of which are associated with monocyte adhesion including *Itgb3*, *Spp1*, and *SelE*. The effects of Cpd71 on *Spp1/SelE/Itgb3* gene expression were validated and these changes were abrogated by OGM‐1, with ICAM gene expression remaining unchanged (Figure 5C). Notably, Spp1 transcript levels were robustly induced by GPR68 agonist treatment (∼3‐fold increase), and this induction was reduced by approximately 50% upon co‐treatment with the GPR68 inhibitor OGM‐1, demonstrating GPR68‐dependent regulation. Further GSEA enrichment of hallmark pathway analysis revealed angiogenesis as the top hallmark pathway (Figure S5A), and angiogenesis GSEA enrichment analysis based on the overlapping genes from “DMSO vs. C71” and “C71 vs. OGM1+C71” comparison showed *Spp1* was ranked top target for GPR68 signaling downstream target (Figure S5B). Consistent with these findings, immunofluorescence analysis showed that Cpd71 treatment significantly increased protein expression of SPP1 in ECs (Figure 5D).

To confirm that the pro‐arteriogenic effects of Cpd71 are mediated specifically through Gpr68 and not off‐target mechanisms, we performed small interfering RNA (siRNA) knockdown experiments in cultured mouse brain microvascular endothelial cells. Gpr68‐knockdown blocked Cpd71 treatment‐induced adhesion of THP‐1 monocytes to ECs as well as significantly inhibited the expression of key arteriogenic adhesion molecules, including *Spp1* and *Itgb3* (Figure S6A–E). These findings provide genetic confirmation that Cpd71 acts as an on‐target agonist of endothelial Gpr68 to drive the inflammatory‐remodeling program.

Taken together, these in vitro results demonstrate that pharmacological activation of GPR68 fosters a pro‐inflammatory EC phenotype conducive to monocyte adhesion.

### 2.7 Systemic Treatment With a GPR68 Agonist Improves Outcomes in HLI

**FIGURE 6:**
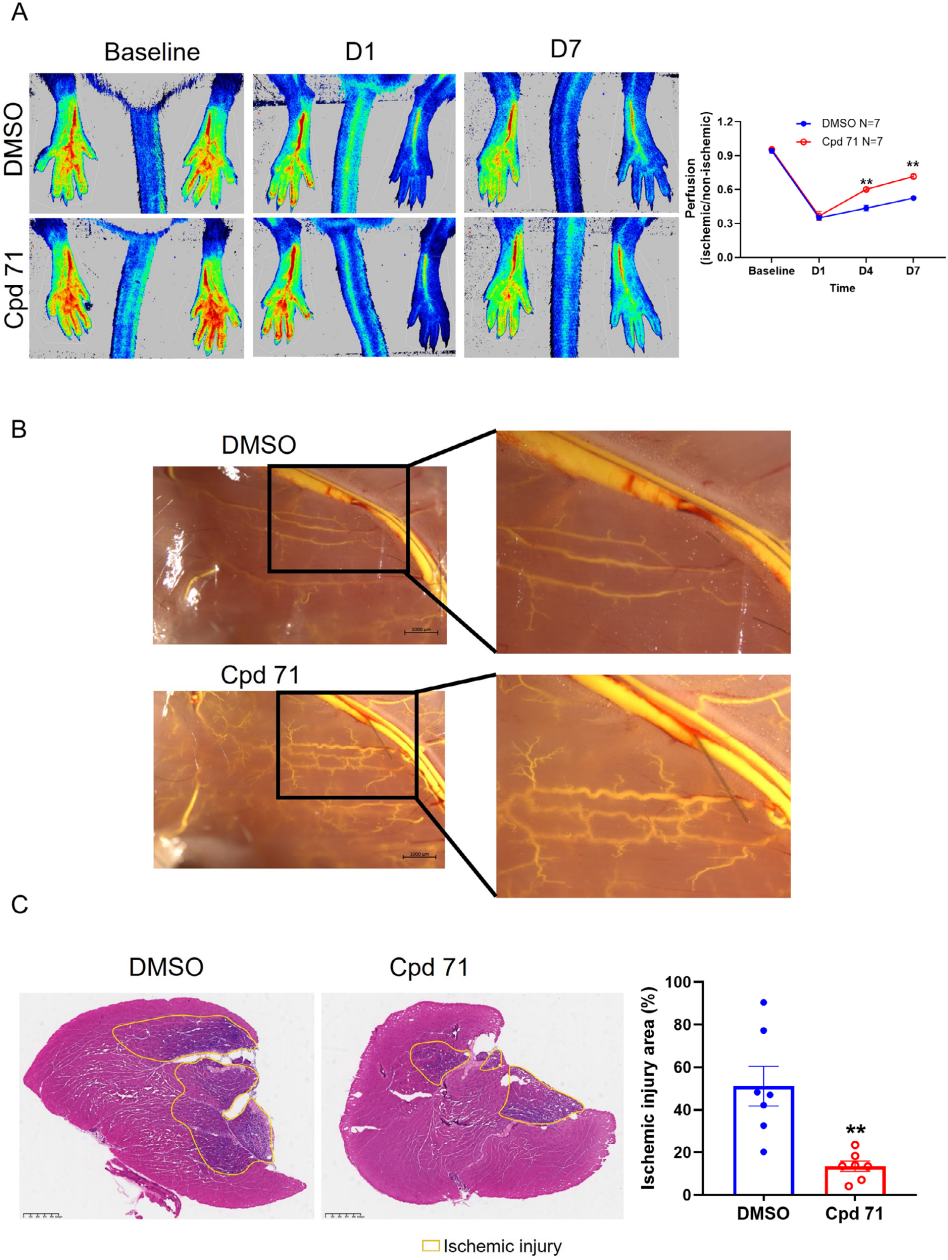
Systemic treatment with a Gpr68 agonist accelerates perfusion recovery and reduces tissue injury in vivo. (A) Representative laser Doppler images and quantification of hindlimb perfusion in HLI mice treated daily with either vehicle (DMSO) or the Gpr68 agonist Compound 71 (Cpd71). Cpd71 treatment significantly accelerated perfusion recovery at days 4 and 7. N = 7 mice per group, **p < 0.01 vs Control, analyzed by Student's t‐test. (B) Representative images of hindlimb collateral vasculature visualized by MICROFIL casting, showing a more robust and extensive collateral network in Cpd71‐treated mice compared to the DMSO group. (C) Representative H&E‐stained cross‐sections of the gastrocnemius muscle and quantification of the ischemic injury area 7 days post‐HLI. The injury area was significantly reduced in the Cpd71‐treated group. N = 7 mice per group, **p < 0.01 vs Control, analyzed by Student's t‐test. Data are presented as mean ± SEM.

Finally, we assessed the therapeutic efficacy of Cpd71 in our in vivo HLI model. Mice treated daily with Cpd 71 (25 mg/kg, i.p.) exhibited significantly accelerated perfusion recovery at both day 4 and day 7 compared to DMSO‐treated controls (Figure 6A). Vessel casting revealed robust collateral networks in Cpd71‐treated mice compared to DMSO group (Figure 6B). Notably, the 10% DMSO vehicle treatment reduced collateral arteriogenesis compared to untreated controls in vivo (Figure 2E), indicating possible vehicle toxicity. Consistent with the relatively improved perfusion, the area of ischemic injury in the gastrocnemius muscle was significantly reduced by 70% in the Cpd71 group compared to DMSO (Figure 6C). Importantly, Cpd71 did not improve perfusion recovery in endothelial‐specific Gpr68 knockout mice (Figure S7), providing strong genetic evidence that the in vivo benefit of Cpd71 is endothelial Gpr68–dependent and supporting an on‐target mechanism of action. These findings demonstrate that systemic pharmacological activation of Gpr68 provides a functional therapeutic benefit, enhancing revascularization and protecting tissue from ischemic damage.

## 3 Discussion

Our findings identify GPR68 as a genetic and mechanistic contributor to peripheral artery disease (PAD), and endothelial GPR68 as a therapeutic target of collateral arteriogenesis after arterial occlusion. Human genetic analysis using UK Biobank data revealed that multiple non‐coding variants across the GPR68 locus are associated with PAD‐related phenotypes, with the strongest signal arising from an upstream regulatory region. While these associations do not establish causality or define functional directionality, they nominate GPR68 as a PAD‐relevant locus and provide a genetic rationale for mechanistic investigation. Notably, the absence of coding variation suggests that disease susceptibility is more likely mediated through context‐dependent regulation of GPR68 expression or signaling rather than altered receptor structure. In the mouse HLI model, inducible endothelial cell‐specific deletion of Gpr68 demonstrates that endothelial Gpr68 is essential for adaptive collateral arteriogenesis. Consequently, the loss of this receptor impairs blood flow restoration, which in turn exacerbates distal muscle injury. Importantly, Gpr68 agonist Cpd71 therapy after HLI showed promising efficacy in blood perfusion restoration and distal muscle injury improvement.

Mechanistically, endothelial Gpr68 promotes monocyte recruitment to remodeling collaterals by enabling adhesion to the endothelial surface and subsequent transmigration into the perivascular space, a defining initiation step in arteriogenesis [20, 28]. In endothelial‐specific Gpr68 knockout (iECKO) mice, peri‐collateral CCR2^+^ monocytes/macrophages were markedly reduced, accompanied by coordinated downregulation of chemokines, adhesion‐related genes, and TNFα‐associated signaling in the collateral bed, as revealed by bulk RNA sequencing and confirmed by reduced Ccl2 and TNFα expression by qPCR. These findings position GPR68 upstream of a pro‐adhesive, endothelial inflammatory program required for monocyte recruitment and collateral growth.

Inflammation in PAD is inherently context‐dependent, driving atherosclerotic plaque progression while being essential for ischemia‐induced collateral remodeling [29]. These processes occur in distinct vascular compartments, with arteriogenesis arising in pre‐existing collateral arterioles exposed to elevated shear stress after arterial occlusion [20, 21]. Consistent with this framework, endothelial Gpr68 activation induced chemokine and adhesion pathways, including Spp1, a marker associated with reparative, pro‐remodeling macrophage function. Although macrophage polarization was not directly assessed, our data support a model in which Gpr68‐mediated inflammation is adaptive in ischemic vascular remodeling, while its role in atherosclerotic disease progression warrants further investigation.

Furthermore, because Gpr68 is known to function as a proton‐sensing G protein‐coupled receptor in the context of tissue ischemia (e.g., in the brain) [19, 30], we considered whether local acidosis might contribute to its activation in the hindlimb. However, a key distinction exists between the ischemic zone (distal limb), where metabolic byproducts and protons accumulate due to poor perfusion, and the collateral zone (proximal limb), where our study was focused. In the collateral‐bearing muscles, the rapid diversion of blood flow following femoral artery occlusion creates a high‐velocity environment [20, 21]. While transient pH fluctuations may occur immediately following ligation, the dominant and sustained stimulus in these remodeling vessels increases fluid shear stress. Our findings suggest that in the peripheral vasculature, Gpr68 is uniquely positioned to sense these mechanical changes, distinct from its role as a pH sensor in the static environment of a cerebral infarct.

Our results align with and extend established roles of Gpr68 as a therapeutic mechanosensor in the vasculature [17, 18]. Our prior work shows that Gpr68 is highly expressed in resistance‐artery endothelium and is required for flow‐mediated Ca^2^^+^ signaling, dilation, and outward remodeling, linking hemodynamic forces to mesenteric vascular adaptation [17]. In the current study, we observed that endothelial Gpr68 orchestrates monocyte recruitment during collateral arteriogenesis in mouse HLI via chemokine/adhesion regulation. Although GPR68 has been implicated in NO‐dependent vasodilation, the use of late time points, fixed‐tissue morphometry, and vascular casting in this study supports a primary role in structural, monocyte‐dependent arteriogenesis, while acknowledging that acute vasodilatory contributions cannot be fully excluded. This provides a solid foundation for pursuing translational potential of Gpr68 in PAD disease settings. Consistent with the requirement of Gpr68 for normal monocyte recruitment and arteriogenesis, pharmacological activation of Gpr68 is sufficient to enhance a pro‐adhesive EC phenotype in vitro and to improve disease outcomes in vivo. The allosteric agonist Cpd71 increased THP‐1 adhesion and induced monocyte‐adhesion transcripts (e.g., *Spp1*, *SelE*, *Itgb3*), effects that were abrogated by a Gpr68 inhibitor and Gpr68 genetic knockdown, supporting an on‐target mechanism of action. Remarkably, daily Cpd71 administration accelerated perfusion recovery, augmented collateral networks, and reduced ischemic injury. Significant medicinal chemistry work is still required to optimize the compound for better bioavailability, pharmacokinetics, absorption‐distribution‐metabolism‐excretion (ADME) properties, as well as formulation, to advance it into the late‐preclinical stage. Nonetheless, our study demonstrates significant therapeutic potential for GPR68 activation in ischemic revascularization in PAD conditions.

This study has several limitations. While this study focuses on mouse endothelial systems, the high conservation of GPR68 supports translational relevance, although validation in human endothelial cells will be required to fully establish clinical applicability. However, GPR68 expression is highly specific to the endothelium of small resistance arteries, a vascular environment characterized by high fluid shear stress and pressure. In contrast, the receptor is notably absent in the endothelial cells of large conduit vessels and the capillary network. Consequently, the choice of cell models for in vitro assays is severely limited, as standard models such as HUVECs or bulk‐isolated hindlimb endothelial cells naturally lack functional GPR68 expression. Compound 71 was used as a pharmacological tool with incomplete dose–response and pharmacokinetic characterization, and the use of a DMSO‐containing vehicle may confound vascular effects despite consistent vehicle controls. Evaluation of Gpr68‐targeted interventions in disease‐relevant models with impaired collateral recovery, such as diabetic, aged, or hyperlipidemic mice, will be essential to establish translational efficacy and clinical relevance beyond the robust remodeling capacity of C57BL/6 mice. Experiments were conducted exclusively in male mice, limiting generalizability given known sex differences in PAD and vascular remodeling. Analyses were performed at a single late time point, capturing established collateral growth but not the early dynamics of monocyte recruitment and arteriogenic initiation. While our findings strongly support an endothelial role for Gpr68 in flow‐driven arteriogenesis in vivo, future studies employing endothelial‐specific single‐cell transcriptomics, real‐time mechanotransduction assays, and rescue approaches will be helpful to further definitively establish EC‐intrinsic mechanosensing mechanisms.

## 4 Conclusion

Together, convergent human genetic association, cell‐type–specific loss‐of‐function, and pharmacological gain‐of‐function support a model in which endothelial GPR68 integrates ischemic injury and hemodynamic cues to activate a chemokine/adhesion program in vivo that recruits monocytes and drives collateral arteriogenesis, thereby limiting downstream tissue injury. Our data also provide the initial preclinical efficacy of GPR68 agonist in an HLI animal model, and positions GPR68 as a promising target for therapeutic arteriogenesis in PAD.

## 5 Materials and Methods

### 5.1 Animal and Husbandry

All experimental protocols were approved by the Institutional Animal Care and Use Committee of Sun Yat‐sen University (SYSU‐IACUC‐2020‐000563). Mice were housed on a 12‐h light/dark cycle with free access to food and water. Gpr68 global KO, Gpr68‐tdTomato reporter, Gpr68fl/fl, mice were generated on a C57BL/6J background, Cdh5‐CreERT2 mice were purchased from Jackson Laboratory. Genotyping was performed by genomic PCR. In Cdh5‐CreERT2 model, efficient and stable recombination is achieved with a finite tamoxifen pulse followed by a “washout” period. This strategy ensures robust gene deletion while eliminating the potential confounding pharmacological effects of tamoxifen on acute vascular responses [31]. Therefore, to induce Cre‐mediated recombination, mice were fed a tamoxifen‐containing diet (400 mg/kg diet) [32]. Based on an average daily food intake of 3–4 g and a body weight of 20–25 g, this regimen resulted in an estimated daily tamoxifen dosage of approximately 60–65 mg/kg body weight. The medicated diet was replaced every 3 days and kept protected from light to prevent drug degradation. For endothelial inducible knockout studies, tamoxifen‐containing chow (400 mg/kg diet) starting 4 weeks and continued until 2 weeks before HLI was administered to induce Cre‐recombinase activity. The efficiency of Cre induction was confirmed using tdTomato mice (Figure S8). All genotype comparisons were performed between littermate controls and knockout mice that were exposed to identical tamoxifen‐containing chow regimens. Therefore, any potential off‐target effects of tamoxifen are equally present in both groups and do not confound the interpretation of genotype‐dependent differences.

### 5.2 Experimental Design and SAGER Guidelines

In accordance with SAGER (Sex and Gender Equity in Research) guidelines, we report that this study was conducted exclusively in male mice aged 12–16 weeks. This single‐sex design was chosen to minimize biological variability associated with the estrous cycle in females and to avoid the known confounding vascular effects of female sex hormones in vascular injury models, thereby reducing the total number of animals required to achieve statistical power. We acknowledge that this approach limits the direct generalizability of our findings. The role of Gpr68 in HLI may differ in females, and future studies are warranted to investigate potential sex‐specific mechanisms.

For comparisons between genotypes, littermate controls were used whenever possible. Within each genotype, mice were randomly allocated to either the HLI or sham surgery group. Key outcome assessments, including the analysis of laser doppler data and the histology quantification, were performed by investigators who were blinded to the genotype and surgical group of the animals.

### 5.3 Hindlimb Ischemia (HLI) Model

Mice were anesthetized with 2% isoflurane and maintained on a heating pad. A dual‐ligation HLI model was used. An incision was made below the inguinal ligament to expose the left femoral artery. The artery was ligated first just distal to the deep femoral artery and second at the popliteal artery distal to its bifurcation from the femoral artery. The skin was closed with 5‐0 silk sutures.

### 5.4 Perfusion Measurement

Blood perfusion was measured using a PeriCam PSI laser Doppler perfusion imaging system (Perimed/PeriCam PSI NR) before surgery and at days 1, 4, and 7 post‐HLI. Mice were anesthetized under isoflurane anesthesia (1.5%–2% inhalation). To ensure that the effects of isoflurane [33] did not confound our results or the comparison between groups, we implemented the following controls: (1) Standardized depth and timing: The concentration of isoflurane and the duration of exposure prior to imaging were strictly standardized across all experimental and control groups to ensure a uniform baseline of drug‐induced vasodilation. (2) Thermal stabilization: Animals were placed on a heating pad to maintain a core body temperature of 37°C throughout the procedure, as fluctuations in temperature significantly modulate the vascular response to isoflurane. (3) Relative flux ratio: Our data are presented as a perfusion ratio (Ischemic/Non‐ischemic limb). Since both limbs are exposed to the same systemic concentration of isoflurane, any drug‐induced vasodilation affects both the control and the experimental side equally, thereby normalizing the confounding effect when calculating the recovery percentage. Perfusion of the footpads was quantified, and the ratio of the ischemic (left) to the non‐ischemic (right) limb was calculated to represent perfusion recovery [34].

### 5.5 Histology and Immunofluorescence

At day 7 after surgery, mice were perfused intracardially with HBSS and fixation solution. The entire adductor muscle region and gastrocnemius muscles were harvested, embedded in OCT compound, rapidly frozen, and sectioned at 10 µm [35]. For immunofluorescence staining, frozen sections were blocked with 5% donkey/goat serum, 1% BSA, and 0.1% Triton X‐100 for 30 min at room temperature, and incubated overnight at 4°C with primary antibodies including α‐SMA (1:500, CST, #19245), RFP (1:100, Rockland, 600‐401‐379), F4/80 (1:100, Invitrogen, 14‐4801‐82), CCR2 (1:250, Abcam, ab273050) and CD31 (1:100, Biolegend, 102507). Sections were then washed and incubated with Alexa Fluor–conjugated secondary antibodies for 1 h at room temperature. Nuclei were counterstained with DAPI. Images were acquired using either a fluorescence microscope (Olympus BX63) or a confocal microscope (Olympus FV3000). Specifically, collateral morphometry was performed from 3 sections of the center part of semimembranosus muscle of the adductor muscle series as it is a major component of the medial thigh/adductor compartment where pre‐existing arterioles reliably undergo outward remodeling into functional collateral arteries [22]. Collateral luminal area and CCR2^+^ cells located within a 30 µm radius from the outer boundary of the vessel wall were counted using OlyVIA 3.3 software. Endothelial (CD31) and smooth muscle (α‐SMA) markers were used to accurately delineate vascular structures and identify remodeling collateral arteries. All image acquisition and quantitative analyses were performed by an investigator blinded to group allocation. For ischemic injury assessment, H&E‐stained sections of the gastrocnemius were imaged [35]. The cumulative area of muscle necrosis, degeneration, and leukocyte infiltration was quantified using ImageJ and expressed as a percentage of the total muscle area.

### 5.6 Identification and Characterization of Collateral Arterioles

To ensure consistent quantification of arteriogenesis, vascular analysis was strictly restricted to the gracilis collateral circui located within the semimembranosus muscle. This anatomically defined pathway connects the deep femoral artery to the saphenous artery. Remodeling collateral arterioles were identified and distinguished from non‐remodeling resistance arterioles in the surrounding tissue based on specific morphological criteria; specifically, collateral vessels were defined by an increased luminal diameter and a characteristic tortuosity (corkscrew appearance) following femoral artery ligation.

### 5.7 Histological Scoring of Muscle Ischemic Injury

Muscle ischemic injury was evaluated using a semi‐quantitative histological scoring system adapted from a previously published method [36]. Briefly, muscle tissues were harvested, fixed, embedded in optimal cutting temperature (OCT) compound, sectioned, and stained with hematoxylin and eosin (H&E) staining. The entire cross‐section area of the gastrocnemius muscle was quantified. Histopathological changes, including inflammation, fibrosis, necrosis, adipocyte infiltration, muscle fiber degeneration/regeneration, and hemorrhage, were assessed. Each parameter was scored on a scale of 0–3 based on severity (0 = normal, 3 = severe) according to established histological criteria.

### 5.8 Vascular Casting

To visualize the collateral vasculature, mice were injected with heparin (100 U, i.p.). Following euthanasia, the abdominal aorta was catheterized, and 200 µL of yellow MICROFIL compound (Flow Tech, Inc.) was perfused [37]. After the compound solidified, hindlimbs were harvested and imaged under bright‐field illumination using a stereomicroscope (Axio Zoom V16, Zeiss).

### 5.9 RNA

Total RNA was extracted from semimembranosus muscle tissue using RNAiso Plus (TAKARA, 9109). RNA seq reads were mapped to the UCSC mm39 (GRCm39) reference genome using HISAT2 (v2.1.0) and gene level counts were summarized using Ensembl annotations. Differentially expressed genes were identified using DESeq2 (version 1.42.1). Gene Set Variation Analysis (GSVA) was performed using the GSVA R Package (version 1.50.5). Per‑sample GSVA scores were compared between groups using linear models with empirical‑Bayes moderation (limma, version 3.58.1), and multiple testing was controlled by Benjamini–Hochberg. Gene sets with *p* value < 0.05 were considered differentially enriched. Gene Set Enrichment Analysis (GSEA) was performed with the fgsea R package (version 1.28.0). Pathways with *p* value < 0.05 were considered significant and the leading‑edge subset was defined at the enrichment score maximum. The GOBP gene sets and the 50 Hallmark Pathways gene sets were obtained from the MsigDB (https://www.gsea‐msigdb.org/gsea/msigdb). To visualize the relationship between pathways and their constituent genes, a pathway–gene network was constructed. Genes belonging to the same enriched pathway were grouped together and connected to the pathway it belongs to. The network visualization was generated using Cytoscape (version 3.10.4). Yellow and green nodes represent genes and pathways, respectively, and edges denote their membership. Genes from the same pathway were clustered together to form functional modules, highlighting key genes driving pathway‐level alterations. For validation, cDNA was synthesized using the PrimeScript RT Reagent Kit (TAKARA, RR047A), and qPCR was performed using TB Green Premix Ex Taq II (TAKARA, RR820A). Gene expression was normalized to 18S rRNA using the ΔΔCT method. The specific primer sequences used for mRNA expression analysis are listed in Table 1.

**TABLE 1:** Primer used in the current study.

Primer	Sequences
*m‐Gpr68‐F*	*ATGAGCTGGGAGTGTACCTGT*
*m‐Gpr68‐R*	*GGAAGCCCACGCTGATGTAAAT*
*18S‐F*	*GCCGCTAGAGGTGAAATTCTT*
*18S‐R*	*CGTCTTCGAACCTCCGACT*
*m‐Itgb3‐F*	*GTGAGTGCGATGACTTCTCCTG*
*m‐Itgb3‐R*	*CAGGTGTCAGTGCGTGTAGTAC*
*m‐Spp1‐F*	*CGTCCCTACAGTCGATGTCC*
*m‐Spp1‐R*	*GGACTCCTTAGACTCACCGC*
*m‐Icam‐F*	*AAACCAGACCCTGGAACTGCAC*
*m‐Icam‐R*	*GCCTGGCATTTCAGAGTCTGCT*
*m‐Vcam‐F*	*GCTATGAGGATGGAAGACTCTGG*
*m‐Vcam‐R*	*ACTTGTGCAGCCACCTGAGATC*
*m‐Mmp9‐F*	*CAGACGTGGGTCGATTCCAA*
*m‐Mmp9‐R*	*CGCGGCAAGTCTTCAGAGTA*
*m‐Nos3‐F*	*CGCAAGAGGAAGGAGTCTAGCA*
*m‐Nos3‐R*	*TCGAGCAAAGGCACAGAAGTGG*
*m‐SelE‐F*	*GGACACCACAAATCCCAGTCTG*
*m‐SelE‐R*	*TCGCAGGAGAACTCACAACTGG*
*m‐Tnfα‐F*	*GATCGGTCCCCAAAGGGATG*
*m‐Tnfα‐R*	*TGAGGGTCTGGGCCATAGAA*
*m‐Ccl2‐F*	*GCTACAAGAGGATCACCAGCAG*
*m‐Ccl2‐R*	*GTCTGGACCCATTCCTTCTTGG*

### 5.10 Cell Culture and In Vitro Assays

The mouse brain endothelial cell line bEnd.3 (RRID:CVCL_0170) was obtained from ATCC (CRL‐2299), purchased from Guangzhou Xinyuan Technology Co., Ltd. (China), and the human monocytic cell line THP‐1 was obtained from the Cell Bank of the Chinese Academy of Sciences (SCSP‐567, RRID: CVCL_0006, Shanghai, China). Authentication: verified by STR profiling prior to use. Contamination status: Supplier provided documentation demonstrating negative results for bacterial and mycoplasma contamination. Both cell lines were used for in vitro experiments. For the adhesion assay, bEnd.3 cells were grown to confluence, serum‐starved, and pre‐treated with Compound 71 (30 µm, MedChemExpress, HY‐134494) or vehicle for 6 h. Calcein‐AM (Invitrogen, L3224) labeled THP‐1 monocytes were then added to the bEnd.3 monolayers for 2 h. Non‐adherent cells were washed away, and the number of adherent monocytes was quantified by microscopy.

For calcium imaging [17], cells loaded with Fluo‐8 (2 µm, AAT Bioquest, 21080) were imaged during treatment with Compound 71 (30 or 50 µm) or vehicle, and the relative change in fluorescence (ΔF/F0) was calculated. Cell migration was assessed via a scratch‐wound assay using a 200 µL pipette tip, with wound closure imaged at 0 and 24 h. For Ki‐67 staining, bEnd.3 cells were stimulated with 30 µm Compound 71 for 6 h and fixed with 4% paraformaldehyde. After washing with PBS, cells were permeabilized and blocked, followed by incubation with an anti–Ki‐67 primary antibody (Cell Signaling Technology, #9129S) at 4°C overnight. Cells were then incubated with a fluorescent secondary antibody, counterstained with DAPI, and imaged by fluorescence microscopy.

### 5.11 siRNA Transfection

Small interfering RNAs (siRNAs) targeting mouse Gpr68 were synthesized by Suzhou GenePharma Co., Ltd. Cells were transfected with siRNAs at a final concentration of 50 nm using RNAiMAX. Briefly, siRNAs and transfection reagent were diluted in Opti‐MEM and added to the cells. After 6 h, the medium was replaced with fresh complete medium, and cells were cultured for subsequent experiments. A scrambled siRNA was used as a negative control. Knockdown efficiency was evaluated by quantitative real‐time PCR (qPCR). Relative Gpr68 expression levels were normalized to an internal control gene and calculated using the 2^‐ΔΔCt method. Small interfering RNAs (siRNAs) targeting mouse Gpr68 were designed and synthesized by Suzhou GenePharma Co.,Ltd. The target sequences were as follows:

1) Gpr68‐Mus‐911: sense 5'‐GUGGCAUCCUCCUCUAUGATT‐3', antisense 5'‐UCAUAGAGGAGGAUGCCACTT‐3'.
2) Gpr68‐Mus‐817: sense 5'‐GCAGACCUGUUCUAUAUCUTT‐3', antisense 5'‐AGAUAUAGAACAGGUCUGCTT‐3'.
3) Gpr68‐Mus‐787: sense 5'‐CUGGGAGUGUACCUGUGUATT‐3', antisense 5'‐UACACAGGUACACUCCCAGTT‐3'.
4) Gpr68‐Mus‐1130: sense 5'‐GAGUCUGCUUUGAGCAUUATT‐3', antisense 5'‐UAAUGCUCAAAGCAGACUCTT‐3'.

### 5.12 Phenome‐Wide Association Study (PheWAS) Analysis of GPR68

Phenome‐Wide Association Study (PheWAS) was conducted using data from the UK Biobank (UKB), a population‑based cohort of ∼500 000 participants aged 40–69 at recruitment [38]. Inclusion was restricted to unrelated individuals with high‐quality genotype data. Participants with genotype‐sex discordance, aneuploidy, high heterozygosity, and missing rate were excluded. Relatedness was handled by excluding one of each related pair. A total of 382 358 quality‐controlled individuals were selected for PheWAS. UKB samples were genotyped and imputed on GRCh38. Variants were filtered to minor allele frequency (MAF) > 0.005, and missingness < 0.02 using Plink 2.0. The GPR68 locus was defined as the transcribed region plus a ±2 kbp window. A total of 295 variants within this window meeting QC criteria were used for locus‐wide analysis. PheWAS analyses were performed using R version 4.0.2. ICD‐10 codes were converted to associated 1387 Phecodes with the PheWAS R package (version 0.99.6.1). Logistic regression was used to assess the association between GPR68 variants and phenotypes, adjusting for age, sex, and ethnic and the 1–20 PCs. The Bonferroni correction method was applied to account for multiple testing, with statistical significance set at p < 0.05. The Phenome‐Wide Association Study (PheWAS) analysis of GPR68 was conducted using the UK Biobank Resource under application number 101596. The UK Biobank data are available on application to the UK Biobank (www.ukbiobank.ac.uk), re‐used with the permission of the NHS England and/or UK Biobank.

### 5.13 Pharmacological Treatment

The GPR68 agonist Compound 71 [19] (MedChemExpress, HY‐134494) was dissolved in DMSO for in vitro use. For in vivo studies, it was formulated in a vehicle of 10% DMSO, 20% SBE, and 70% saline. Mice received daily intraperitoneal injections of either Compound 71 (25 mg/kg) or vehicle for seven days, starting one day after HLI surgery.

### 5.14 Statistical Analysis

All quantitative data are presented as mean ± SEM. Statistical analyses were performed using GraphPad Prism software (v9.0, GraphPad Software). The Shapiro‐Wilk test was used to assess normality for all datasets. For comparisons between two groups with normally distributed data, an unpaired, two‐tailed Student's t‐test was used. For comparisons between more than two groups with normally distributed data, a one‐way or two‐way Analysis of Variance (ANOVA) was performed, followed by Tukey's multiple comparisons post‐hoc test. For data that were not normally distributed, the non‐parametric Mann–Whitney U test (for two groups) or the Kruskal–Wallis test with Dunn's multiple comparisons test (for multiple groups) was used. A *p*‐value < 0.05 was considered statistically significant. The specific tests used for each experiment are detailed in the figure legends.

## Funding

This study was supported by the Natural Science Foundation of China (32371199, 82270274, 82070311), the Guangdong Provincial Science and Technology Department (2023ZT10Y154), and the Chinese National Key Research and Development Program (2023YFC2307004).

## Conflicts of Interest

The authors declare no conflicts of interest.

## Supporting information




**Supporting File 1**: advs74895‐sup‐0001‐SuppMat.docx.

**Supporting File 2**: advs74895‐sup‐0001‐FigureS1‐TIF.

**Supporting File 3**: advs74895‐sup‐0002‐FigureS1‐TIF

**Supporting File 4**: advs74895‐sup‐0003‐Data.zip.

**Supporting File 5**: advs74895‐sup‐0004‐Data.zip.

**Supporting File 6**: advs74895‐sup‐0005‐Data.zip.

**Supporting File 7**: advs74895‐sup‐0006‐Data.zip.

**Supporting File 8**: advs74895‐sup‐0007‐Data.zip.

**Supporting File 9**: advs74895‐sup‐0008‐Data.zip.

**Supporting File 10**: advs74895‐sup‐0010‐Data.zip.

## Data Availability

The data that support the findings of this study are available from the corresponding author upon reasonable request.
