# Supplementary material for: Endothelial GPR68 Is Essential for Arteriogenesis and Represents a Therapeutic Target in a Model of Peripheral Artery Disease
Source: Adv Sci (Weinh). 2026 Mar 20;13(31):e20244. doi: 10.1002/advs.202520244 (PMC13587936; doi:10.1002/advs.202520244)
Supplement: Supplementary file 1 [file ADVS-13-e20244-s003.docx]

***Supplemental Figure Legends***

**Supplemental Figure 1**. Expression of GPR68 following hindlimb ischemia and baseline gene expression in Gpr68 iECKO mice.

(A) Phenome-wide association study (PheWAS) plot (shape coded) showing associations between variants in the GPR68 locus and various disease categories from the UK Biobank. The y-axis represents the odds ratio (OR), and points are colored by disease group, with circulatory system traits highlighted in blue circle. The top three associated circulatory system diseases are: 1: Chronic vascular insufficiency of intestine; 2: Disease of capillaries; 3: Atherosclerosis of native arteries of the extremities with intermittent claudication (B) Schematic of the Gpr68-tdTomato reporter mouse allele, where a tdTomato fluorescent protein sequence is inserted in-frame before the stop codon of the Gpr68 gene. (C) Representative immunofluorescence images of collateral arterioles in the semimembranosus muscle of the adductor muscle series from Gpr68-tdTomato reporter mice. Images show GPR68 expression (red, tdTomato signal), endothelial cells (green, CD31), smooth muscle cells (white, αSMA) and nuclei (blue, DAPI) 7 days after HLI. The merged image shows specifically co-localization of Gpr68 in endothelial cells of arterioles, not in the veins and capillaries. (D) Immunofluorescence staining for macrophages (F4/80, green) and Gpr68 (RFP/tdTomato, red) in the semimembranosus muscle (SM). Seven days after HLI (Ligated-SM), there is a significant infiltration of Gpr68+ macrophages around collateral vessels compared to the unligated limb. (E) Staining for neutrophils (LY6G, green) in the ligated SM shows no significant neutrophil presence around collateral arteries 7 days post-HLI. (F) In the ischemic gastrocnemius muscle (GC), Gpr68+ cells (red) increase after ligation and co-express the pan-leukocyte marker CD45 (green). Nuclei are counterstained with DAPI (blue). Scale bars = 50 µm in (C), 100 µm in (D-F).

**Supplemental Figure 2.** Endothelial Gpr68 Deficiency and Baseline Vascular Markers

(A) Representative image of arteriogenesis and endothelial markers: A semimembranosus muscle section from a mouse 7 days after hindlimb ischemia (HLI) showing the co-localization of smooth muscle actin (αSMA, green) and CD31 (red). Scale bar = 50 µm. (B) Baseline collateral tortuosity: Representative bright-field images of the collateral vasculature in Control and Gpr68 iECKO mice at baseline, showing no significant morphological differences before injury. (C) Perivascular macrophage identity: Multi-channel immunofluorescence images of a remodeling collateral artery 7 days post-HLI. Staining includes DAPI (nuclei, blue), SMA (smooth muscle, white), CD68 (macrophage marker, green), and CCR2 (recruited monocyte marker, red). The merged image confirms the presence of perivascular CCR2+ macrophages. Scale bar = 50 µm. (D) Gene expression in adductor tissue 7 days after HLI: Quantitative PCR (qPCR) analysis showing the relative mRNA levels of adhesion and inflammatory markers—*Icam*, *Vcam*, *Mmp9*, and *Nos3*—in the collateral-containing muscle. N=5 mice per group, no significant differences (n.s) vs Control, analyzed by Student’s t-test. (E) qPCR analysis demonstrating significant reduction of Gpr68 mRNA in Gpr68 iECKO and Gpr68ΔMac mice. N=4-5 mice per group, * *P*<0.05 vs Control, analyzed by Student’s t-test. Data are presented as mean ± SEM.

**Supplemental Figure 3.** Characterization of GPR68 expression and agonist specificity in cultured endothelial cells.

(A) Immunofluorescence image of primary cultured mouse brain endothelial cells (ECs) stained for the endothelial marker CD31 (red) and DAPI (blue). (B) Primary ECs from Gpr68-tdTomato mice showing co-localization of GPR68 (green, tdTomato) and CD31 (red). (C) Calcium imaging in primary ECs from wild-type (WT) and Gpr68 global knockout (KO) mice. Representative traces show that the GPR68 agonist Compound 71 (Cpd71) induces a robust intracellular Ca²⁺ transient (ΔF/F₀) only in WT ECs, confirming its target specificity. (D) Conformation of Gpr68 mRNA knockdown in primary EC isolated from WT and Gpr68 global KO mice. N=4 mice per group, ***P* < 0.01 vs Control, analyzed by Student’s t-test. (E) Calcium imaging in a mouse brain microvascular endothelial cell (bEnd.3) line confirms functional GPR68 expression, as shown by the Ca²⁺ response to Cpd71 (30µM and 50 µM) stimulation. (F) Calcium imaging in HUVEC line confirms no functional GPR68 expression, as shown by lack of the Ca²⁺ response to Cpd71 (50 µM) stimulation. Scale bars = 200 µm (A), 50 µm (B).

**Supplemental Figure 4.** Gpr68 agonist Compound 71 does not affect endothelial cell proliferation, migration, or viability.

(A-B) Representative images (A) and quantification (B) of Ki67 staining (pink) in endothelial cells treated with DMSO or Compound 71 (Cpd71), showing no effect on cell proliferation. Nuclei are stained with DAPI (blue). Representative images (C) and quantification (D) of a scratch-wound healing assay, demonstrating that Cpd71 does not alter the migration or recovery ratio of endothelial cells compared to the DMSO control. (E) Lactate dehydrogenase (LDH) release assay shows that Cpd71 is not cytotoxic to endothelial cells at the concentrations used in experiments. N=3-6 independent experiments per group, n.s: no significant difference vs Control analyzed by Student’s t-test. Data are presented as mean ± SEM. Scale bars = 100 µm (A), 500 µm (D).

**Supplemental Figure 5**. Gene‑set enrichment analysis (GSEA) of Hallmark pathways

(A) Bubble plot summarizing Hallmark pathway enrichment across two contrasts. The x‑axis shows the enrichment score (ES) for Cpd 71 (C71) vs OGM‑1; the y‑axis shows the negated ES for OGM‑1 vs C71, such that pathways in the upper‑right quadrant are positively enriched in C71 and display the reciprocal trend in the reverse comparison. Each dot represents one Hallmark pathway. Dot color encodes statistical significance for OGM‑1 vs C71 (–log10 P; higher values in red), and dot area encodes significance for C71 vs DMSO (–log10 P; larger circles indicate greater significance). Representative pathways of interest (e.g., Angiogenesis, Coagulation, Myogenesis) are labeled. (B) GSEA output based on the overlapping genes from “DMSO vs. C71” and “C71 vs. OGM1+C71” comparison for HALLMARK_ANGIOGENESIS. The running enrichment curve is plotted across the ranked gene list; vertical tick marks (“barcode”) indicate the positions of angiogenesis‑set members, and labeled genes denote the leading‑edge subset (e.g., *Itgav*, *Jag1*, *Fgfr1*, *Spp1*, *Vegfa*, *Pdgfa*, and *Stc1*). The heat map below displays selected angiogenesis genes (rows) across individual samples (columns) from the three treatment groups. Expression values are centered and scaled per gene (row z‑score; scale –1.5 to 1.5). The top color strip indicates sample groups: DMSO (blue), C71 (yellow), and OGM‑1 + C71 (red).

**Supplemental Figure 6.** GPR68 knockdown abolishes Compound 71-induced monocyte adhesion and pro-inflammatory gene expression in endothelial cells.

(A) Representative fluorescence images (left) and quantification (right) of THP-1 monocyte adhesion to mouse brain microvascular endothelial cells transfected with scrambled siRNA (si-Scrambled) or Gpr68-targeting siRNA (si-Gpr68). Endothelial cells were treated with vehicle (DMSO) or the GPR68 agonist Compound 71 (Cpd71, 30 µM). Green dots represent adherent calcein-AM-labeled THP-1 cells. (B) Quantitative PCR (qPCR) validation of Gpr68 knockdown efficiency in mBMVECs, showing more than 50% reduction in mRNA levels in the si-Gpr68 group compared to si-Scrambled. N=3 independent experiments. ** *P*<0.01 vs si-Scrambled, analyzed by Student’s t-test (C-E) qPCR analysis of downstream adhesion molecule expression, including Spp1 (C), Itgb3 (D), and SelE (E). Data are presented as mean ± SEM. N=3 independent experiments. ***P* < 0.01 vs. DMSO (si-Scrambled); # *P* < 0.05 vs. si-Scrambled analyzed by two-way ANOVA with Tukey's post-hoc test.

**Supplemental Figure 7.** The therapeutic effect of Gpr68 agonist is endothelial Gpr68-dependent.

(A) representative laser doppler perfusion images: Representative images of hindlimb blood perfusion in inducible, endothelial cell-specific Gpr68 knockout (Gpr68 iECKO) mice treated with either vehicle (DMSO) or the Gpr68 agonist Compound 71 (Cpd 71) at 7 days post-hindlimb ischemia (HLI). (B) quantification of blood perfusion recovery: Longitudinal measurement of the ratio of ischemic to non-ischemic limb perfusion from baseline to day 7 post-surgery. N=5 mice per group, n.s: no significant difference vs Control analyzed by Student’s t-test. Data are presented as mean ± SEM

**Supplemental Figure 8.** Validation of Cdh5-Cre-mediated recombination efficiency.

Representative immunofluorescence images of the semimembranosus muscle 7 days after hindlimb ischemia (HLI) from Cdh5-CreERT2;tdTomato mice. To induce endothelial-specific recombination, mice were fed a tamoxifen-containing diet starting at 4 weeks of age and continued until 2 weeks before HLI surgery. Sections are stained for nuclei (DAPI, blue), the reporter for Cre activity (tdTomato, green), and the endothelial marker (CD31, red). The merged image demonstrates robust and specific recombination within the endothelial layer of the remodeling collateral vasculature.
